# Pharmacologic activation of cholinergic alpha7 nicotinic receptors mitigates depressive-like behavior in a mouse model of chronic stress

**DOI:** 10.1186/s12974-017-1007-2

**Published:** 2017-12-02

**Authors:** Dan Zhao, Xulin Xu, Linna Pan, Wei Zhu, Xiaopei Fu, Lianjun Guo, Qing Lu, Jian Wang

**Affiliations:** 1 Department of Pharmacology, School of Basic Medicine, Tongji Medical College, Huazhong University of Science and Technology, Wuhan, 430030 China; 2 Department of Pharmacy, Jiangxi Provincial Cancer Hospital, Nanchang, 330029 China; 3 The Key Laboratory for Drug Target Research and Pharmacodynamic Evaluation of Hubei Province, Wuhan, 430030 China; 4 Department of Neurology, The Second Hospital of Nanchang, Nanchang, 330003 China; 5 Department of Emergency Medicine, Tongji Hospital, Tongji Medical College, Huazhong University of Science and Technology, Wuhan, 430030 People’s Republic of China; 6 Department of Anesthesiology and Critical Care Medicine, Johns Hopkins University, School of Medicine, Baltimore, MD 21205 USA; 7 Department of Human Anatomy, Basic Medical College of Zhengzhou University, Zhengzhou, Henan 450001 People’s Republic of China

**Keywords:** Chronic restraint stress, Nicotinic acetylcholine receptor, TLR4, Neuroinflammation, Depression

## Abstract

**Background:**

It has been shown that chronic stress-induced depression is associated with exaggerated inflammatory response in the brain. Alpha7 nicotinic acetylcholine receptors (α7nAChRs) regulate the cholinergic anti-inflammatory pathway, but the role of cholinergic signaling and α7nAChR in chronic stress has not yet been examined.

**Methods:**

In this study, we used a well-documented model of depression in which mice were exposed to 6 h of restraint stress for 21 consecutive days. Components of cholinergic signaling and TLR4 signaling were analyzed in the hippocampus. The main targets of neuroinflammation and neuronal damage were also evaluated after a series of tests for depression-like behavior.

**Results:**

Chronic restraint stress (CRS) induced alterations in components of central cholinergic signaling in hippocampus, including increases in choline acetyltransferase protein expression and decreases in nuclear STAT3 signaling. CRS also increased TLR4 signaling activity, interleukin-1β, and tumor necrosis factor-α expression, microglial activation, and neuronal morphologic changes. Cholinergic stimulation with the α7nAChR agonist DMXBA significantly alleviated CRS-induced depressive-like behavior, neuroinflammation, and neuronal damage, but these effects were abolished by the selective α7nAChR antagonist α-bungarotoxin. Furthermore, activation of α7nAChRs restored the central cholinergic signaling function, inhibited TLR4-mediated inflammatory signaling and microglial activity, and increased the number of regulatory T cells in the hippocampus.

**Conclusions:**

These findings provide evidence that α7nAChR activation mitigates CRS-induced neuroinflammation and cell death, suggesting that α7nAChRs could be a new therapeutic target for the prevention and treatment of depression.

**Electronic supplementary material:**

The online version of this article (10.1186/s12974-017-1007-2) contains supplementary material, which is available to authorized users.

## Background

Chronic mental and emotional stress are detrimental to physical and mental well-being and often cause affective disorders such as depression and anxiety [1]. It has been shown that chronic stress challenges are associated with an inflammatory response in brain that is characterized by toll-like receptor-4 (TLR4)/nuclear factor kappa B (NF-κB) activation and release of proinflammatory cytokines [2, 3].

TLRs are pattern recognition receptors thought to mediate the innate immune response [4, 5]. Their expression is modulated rapidly in response to pathogens, a variety of cytokines, and environmental stress. Specifically, TLR4 regulates the adrenal response to stress and inflammatory stimuli as well as the brain’s response to stress [6]. TLR4 recruits adapter proteins, such as myeloid differentiation factor 88 (MyD88), and activates downstream signaling molecules, such as transcription factor nuclear factor kappa B (NF-κB), to elicit production of multiple proinflammatory cytokines. Activation of TLR4 complex may underlie the pathophysiology of many inflammatory diseases, such as depression, diabetes, obesity, chronic fatigue syndrome, and neuroinflammatory disorders [7]. Drugs and compounds that attenuate the TLR signaling pathway could represent new treatment possibilities for TLR4-mediated inflammatory disorders.

The cholinergic system plays a crucial role in motor, cognitive, and affective processes, and also regulates central and peripheral inflammation [8, 9]. Acetylcholine interacts with nicotinic acetylcholine receptor α7 subunit (α7nAChR) in tissue macrophages and other immune cells and inhibits the synthesis/release of tumor necrosis factor-α (TNF-α) and other inflammatory cytokines. This neural anti-inflammatory response, known as the cholinergic anti-inflammatory pathway, is fast and integrated through the central nervous system (CNS) [8]. α7nAChRs, composed of five identical α7 subunits, mainly regulate the cholinergic anti-inflammatory pathway by mediating peripheral macrophage activity [10]. In the CNS, both microglia and neurons express functional α7nAChRs. Indeed, stimulation of microglial α7nAChRs was shown to inhibit glial activation and decrease proinflammatory mediator expression and reactive oxygen species production [11, 12]. Dysregulation of cholinergic signaling has been implicated in multiple inflammatory disorders, including sepsis, myocardial or cerebral ischemia, cerebral hemorrhage, and Alzheimer’s disease [12–16]. Activation of the cholinergic system after brain injury might repair the CNS capacity to control the exaggerated inflammation.

Neuronal cell damage after chronic stress is observed predominantly in the hippocampus, a structure densely populated with α7nAChRs. Although the TLR4 proinflammatory pathway has been implicated in the development of chronic stress [2, 17], the roles of cholinergic signaling and α7nAChR-associated anti-inflammatory properties in chronic stress have not yet been examined. We therefore investigated whether chronic immobilization stress disrupts cholinergic function and activates the inflammatory responses, and whether treatment aimed at restoring α7nAChR function offers benefits by regulating inflammation and reducing neuronal death.

## Methods

### Animals

Adult male Kunming mice (25 ± 2 g) were obtained from the Center for Experimental Animals, Huazhong University of Science and Technology, China. Animals were housed under standard laboratory conditions with a 12-h light/dark cycle and free access to food and water. All procedures were approved by the Institutional Animal Care and Use Committee at the Huazhong University of Science and Technology and conformed to the National Institutes of Health *Guide for the Care and Use of Laboratory Animals* (approval number S634/2016).

### Chronic restraint stress (CRS) procedure and assessment

**Fig. 1:**
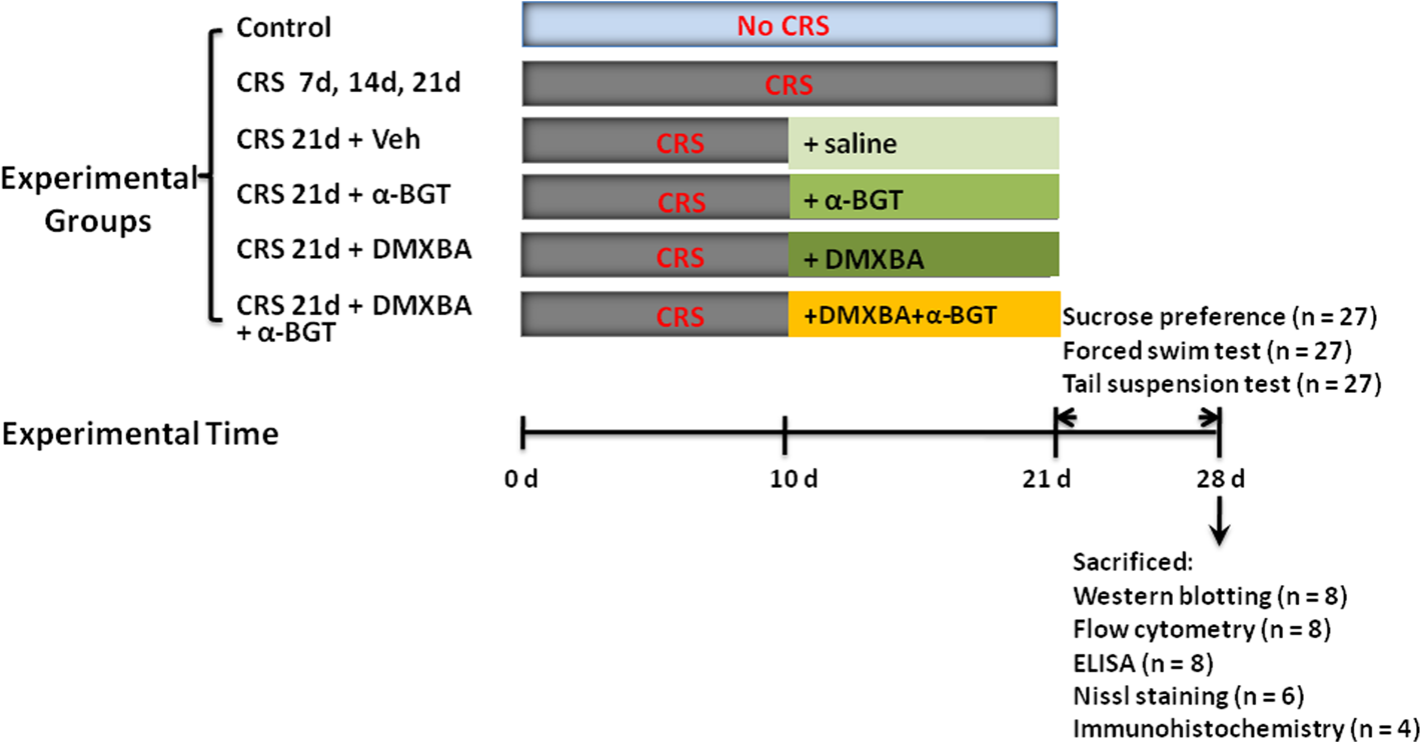
Schematic representation of the experimental design. α-BGT, α-bungarotoxin; CRS, chronic restraint stress

Each mouse was restrained without access to food or water for 6 h per day (10:00 to 16:00) in a well-ventilated 50-mL conical Plexiglas tube for 21 days, as described previously [18]. During the restraint time, control animals were handled and stayed in their home cage without water or food. Animals were returned to their home cages after each period of restraint. An investigator without knowledge of the groups evaluated depressive-like behaviors with the sucrose preference test, tail suspension test, and forced swim test beginning 24 h after the last immobilization exposure. Each behavioral test was carried out on a different day. After behavioral testing was complete, mice were anesthetized with an intraperitoneal injection of ketamine (100 mg/kg, Sigma, St. Louis, MO, USA), blood was collected, and spleens were removed. Simultaneously, brains were removed, and both hippocampal areas were isolated and frozen at − 80 °C until processing. The experimental design is illustrated in Fig. 1.

### Reagents and treatment

DMXBA (Abcam, Cambridge, UK), a selective α7nAChR agonist, was dissolved in saline and administered daily by intraperitoneal injection at a dose of 4 mg/kg, beginning on day 10 of CRS. Peripherally administered DMXBA has a biological half-life of 12–24 h [16] and readily crosses the blood–brain barrier [19]. In a subgroup, mice were pretreated intraperitoneally with α7nAChR antagonist α-bungarotoxin (α-BGT; 1 μg/kg, Abcam) 15 min before DMXBA treatment. A control group of CRS-exposed mice were administered saline. Normal healthy mice with or without saline treatment served as negative controls. The delivery time and doses of DMXBA and α-BGT were chosen on the basis of our preliminary experiments and relevant references [13, 15, 20].

### Forced swim test (FST)

The FST was carried out according to the method described previously [21, 22]. A mouse was placed in a cylinder 20 cm high and 12 cm in diameter containing 10 cm of water (25 ± 1 °C). The FST procedure included two periods: an initial 5-min training session and a 5-min test session conducted after 24 h. The mouse was considered to be (1) immobile if it made no movements, (2) struggling if it dove or tried to climb the wall, and (3) swimming if it made active swimming or circling movements. The total immobility time within the 5-min test was recorded. An increased immobility time was indicative of depressive-like behavior.

### Tail suspension test (TST)

The TST was carried out according to a method described previously [23]. Each mouse was suspended by the tip of its tail with adhesive tape to a hook 50 cm above the floor in a soundproof box. The total immobility time was recorded as the time during which the mouse hung passively and completely motionless within a 5-min test period.

### Sucrose preference test (SPT)

Anhedonia, a key feature of depressive-like behavior, was tested with the SPT as described [23]. Individually caged mice were acclimated to two water bottles, one containing water and the other 1% sucrose solution (1%, *w*/*v*). After baseline preference was determined, mice were deprived of water and food for 22 h and then given free access to the two bottles for 2 h. Sucrose preference was calculated as sucrose consumption/(sucrose consumption + water consumption) × 100%.

### Open field test

One set of mice was used to evaluate the locomotor activity in an open-field test as described previously [21]. The open-field apparatus was made of clear Plexiglas surrounding a 40 × 40 cm open center. The mice were placed individually in the center for 5 min after 11 consecutive days of receiving DMXBA, α-BGT, or saline. The total distance traveled (m) and the average speed (m/s) were recorded under dim light conditions. The instrument was thoroughly cleaned between each test.

### Flow cytometric analysis

Mice in each group were killed under anesthesia and the spleens rapidly removed. Spleens were dispersed in phosphate-buffered saline with the plunger of a syringe and pressed through a cell strainer. Suspensions of splenocytes were then collected by centrifugation (1000 rpm, 10 min). Cell pellets were incubated with fluorescently labeled antibodies to CD4, CD25, and Foxp3 (eBioscience, San Diego, CA, USA) as described previously [24]. Numbers of regulatory T (Treg) cells were measured by flow cytometry (FACSCalibur, Becton Dickinson, San Jose, CA, USA).

### Western blot analysis

Mice in each group were killed under anesthesia and their brains rapidly removed. Hippocampal tissue was cut and prepared for protein analysis as descried previously [25–27]. Cells were lysed in RIPA buffer supplemented with protease inhibitor cocktail (Beyotime, Shanghai, China) on ice. Nucleoproteins were prepared by using NE-PER Nuclear and Cytoplasmic Extraction Reagents (Thermo-Scientific, Franklin, MA, USA). The protein concentration was determined with a BCA kit (Pierce, Woburn, MA, USA). Equal amounts of protein from each group were diluted in loading buffer, separated by 8% sodium dodecyl sulfate-polyacrylamide gel electrophoresis, and then transferred to a polyvinylidene difluoride membrane (Roche, Basel, Switzerland) as previously described [28]. Membranes were blocked with 5% non-fat milk in Tris-buffered saline containing 0.1% Tween-20 for 1 h at room temperature and then incubated overnight with constant agitation at 4 °C with one of the following primary antibodies: anti-TLR4 (1:1000, Abcam), anti-MyD88 (1:200, BOSTER, Wuhan, China), anti-NF-κB (1:1000, Cell Signaling, Boston, MA, USA), anti-IBA-1 (1:1000, Abcam), anti-CHRNA7 (1:1000, Abcam), anti-choline acetyltransferase (ChAT, 1:200, BOSTER), anti-signal transducer and activator of transcription (STAT) 3 (1:1000, Cell Signaling), anti-acetyl-histone H3B (Lys5; 1:1000, Cell Signaling), or anti-GAPDH (1:5000, CWBiotech, Beijing, China). The antigen-antibody complexes were visualized with relevant secondary antibodies by using ECL-Plus (Millipore, Bedford, MA, USA). Protein content was normalized to GAPDH or histone. All results were determined by NIH ImageJ software.

### Acetylcholinesterase (AChE) activity assay

The activity of AChE, the enzyme responsible for synaptic clearance of acetylcholine, was assessed with a commercially available AChE assay kit (Jiancheng Bioengineering Institute, Nanjing, China), as described previously [29, 30]. Samples of 0.1 mL hippocampal tissue homogenate containing 400 μg protein were added to the chemical reaction system. The change in absorbance was measured at 412 nm in a TECAN Infinite M200 PRO microplate reader. AChE activity is expressed as units per milligram protein.

### Immunofluorescence

Immunofluorescence procedures were performed as described previously [31, 32]. Mice were deeply anesthetized and perfused transcardially with 30 mL of 0.9% saline solution (37 °C) followed by 100 mL of 4% paraformaldehyde (Sigma) in 0.1 mol/L phosphate buffer (pH 7.4). Subsequently, brains were removed and kept in 4% paraformaldehyde (Sigma) overnight at 4 °C and then embedded in paraffin. The brains were cut into 5-μm-thick slices, which were incubated with goat anti-Iba-1 (1:500, Abcam), rabbit anti-NeuN (1:200, Millipore), mouse anti-GFAP (1:500, Abcam), or rabbit anti-CHRNA7 (1:200, BOSTER) overnight at 4 °C. Finally, the slices were incubated with the corresponding secondary antibody for 2 h. The sections were analyzed with an Olympus FluoView 1200 confocal microscope system (Olympus Corporation, Tokyo, Japan), and photomicrographs of representative fields were taken.

### Nissl staining

Neuronal damage in brain sections was determined by Nissl staining. Paraffin-embedded tissue was cut into 5-μm-thick sections on a vibratome (Campden Instruments, MA752, Leicester, UK) and was placed onto slides. The slides were subjected to Cresyl violet (Sigma) staining to visualize Nissl substance as described previously [33, 34]. Images of the CA1, CA3, and dentate gyrus regions were acquired by phase contrast light microscopy.

### Golgi staining

After mice were perfused transcardially with 4% paraformaldehyde, the brains were removed and stained by the modified Golgi-Cox method [31, 35]. Coronal sections of 50 μm thickness were obtained with a vibratome (Campden Instruments). The Golgi-impregnated neurons in the hippocampal CA1 region were studied, and dendritic images were acquired on a Zeiss fluorescence microscope with × 4 and × 100 objectives. Investigators blind to experimental conditions counted dendritic spines in CA1 neurons (50-μm segment length on 10 neurons from each mouse) with Image-Pro Express software.

### ELISA assay for cytokine release

Interleukin-1β (IL-1β) and TNF-α levels in hippocampus and peripheral blood plasma were measured with commercially available ELISA kits (Bo Yan Biological Technology Co., Shanghai, China) according to the manufacturer’s instructions [36]. Absorbance at 450 nm was measured on a microplate reader.

### Statistical analysis

Data were analyzed with GraphPad Prism v.5 (GraphPad, San Diego, CA, USA) and expressed as means ± SEM. Differences between means were assessed by one-way ANOVA followed by Bonferroni’s post hoc test between multiple groups. In all comparisons, differences were considered significant at *P* < 0.05.

## Results

### Central cholinergic signaling in the hippocampus is altered in mice after CRS exposure

**Fig. 2:**
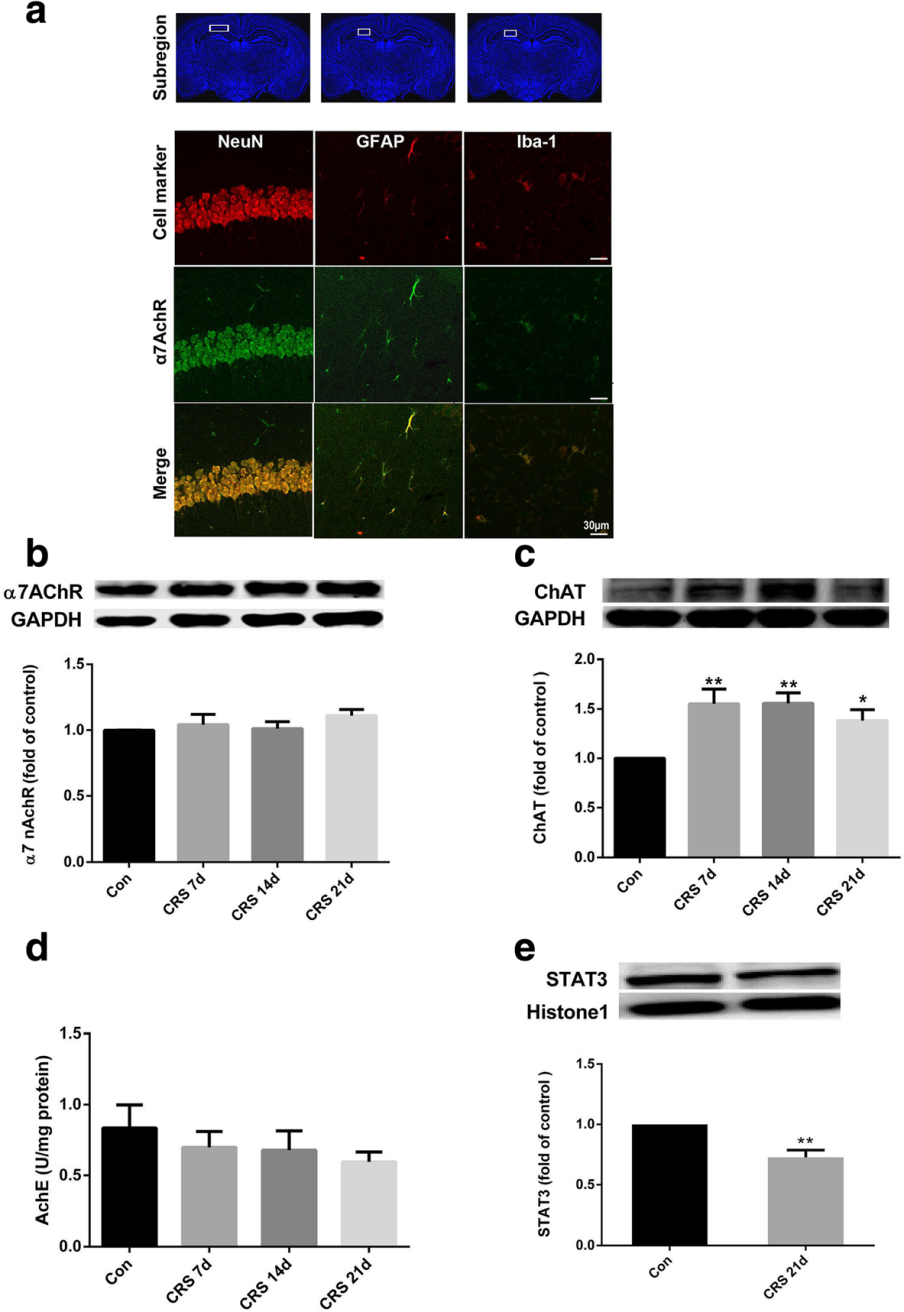
Chronic restraint stress (CRS)-induced alterations in components of central cholinergic signaling in hippocampus. **a** Double-immunolabeling showed that α7nAChR colocalized primarily with NeuN^+^ neurons and to a lesser extent with GFAP^+^ astrocytes and Iba-1^+^ microglia in the hippocampus; scale bar, 30 μm. **b** Representative immunoblots and relative levels of α7nAChR protein in hippocampus. Results are shown as fold change relative to GAPDH protein level. **c** Representative immunoblots and relative levels of choline acetyltransferase (ChAT) protein in hippocampus. Results are shown as fold change relative to GAPDH protein level. **d** Acetylcholinesterase (AChE) activity in hippocampus at different time points of CRS. **e** Representative immunoblots and relative levels of STAT3 protein in hippocampus. Results are shown as fold change relative to histone1 protein level. Con, control group. Data are expressed as mean ± SEM. *n* = 8 per group; *P < 0.05, **P < 0.01 vs. control group

To clarify the cell type that expresses α7nAChR in brain, we performed double-immunolabeling with cell-type-specific antibodies. In the hippocampus, α7nAChR colocalized primarily with NeuN^+^ neurons, and to a lesser extent with GFAP^+^ astrocytes and Iba-1^+^ microglia (Fig. 2a). Stressed mice exhibited increased hippocampal expression of ChAT, the enzyme responsible for acetylcholine synthesis. Additionally, mice displayed a trend toward increased expression of α7nAChR and a trend toward decreased expression of AChE in the hippocampus after 21 days of CRS. However, intranuclear STAT3, the major transcription factor-mediated cytokine signal transduction protein in the cholinergic anti-inflammatory pathway, was significantly reduced after 21 days of CRS compared with that in controls (Fig. 2b–e).

### TLR4 signaling and inflammation are upregulated in the hippocampus after CRS exposure

**Fig. 3:**
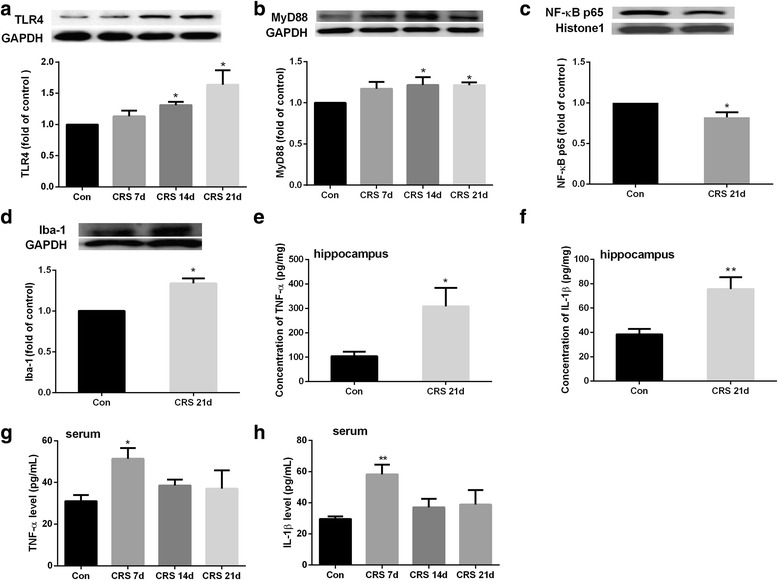
Chronic restraint stress (CRS)-induced inflammatory cytokine over-expression and inflammatory signal activation. Protein levels were assessed at 7, 14, and 21 days of CRS. **a–d** Representative immunoblots and relative levels of TLR4 protein (**a**), MyD88 protein (**b**), NF-κB protein (**c**), and Iba-1 protein (**d**) in hippocampus. Results are shown as fold change relative to GAPDH (**a**, **b**, **d**) or histone 1 (**c**) protein level. **e**–**h** ELISA was used to measure levels of TNF-α and IL-1 in the hippocampus (**e**, **f**) and serum (**g**, **h**). Con, control group. All data are expressed as mean ± SEM. *n* = 8 per group; *P < 0.05, **P < 0.01 vs. control group

We analyzed expression of proteins in the TLR4 pathway to evaluate neuroinflammation after chronic stress (Fig. 3). Expression levels of TLR4 and its downstream adaptor MyD88 were significantly increased in the hippocampus after 14 and 21 days of chronic stress compared with expression in the control mice; however, the protein expression of NF-κB in the nucleus was decreased at 21 days (Fig. 3a–c). Additionally, 21 days of CRS led to elevated microglial activity, as evidenced by increased Iba-1 level in the hippocampus (Fig. 3d). We also detected a significant increase in the proinflammatory cytokines TNF-α and IL-1β at 21 days of CRS, although the expression of nuclear NF-κB p65 subunit was decreased in hippocampus (Fig. 3e, f). However, levels of these cytokines in the hippocampus did not differ significantly from those of the control group at days 7 and 14 of CRS (Additional file 1). Further, serum levels of TNF-α and IL-1β were significantly increased at 7 days of CRS, but were not significantly changed at 14 and 21 days, when compared with levels in the control group (Fig. 3g, h).

### Cholinergic stimulation by the α7nAChR agonist DMXBA ameliorates CRS-induced depression-like behaviors

**Fig. 4:**
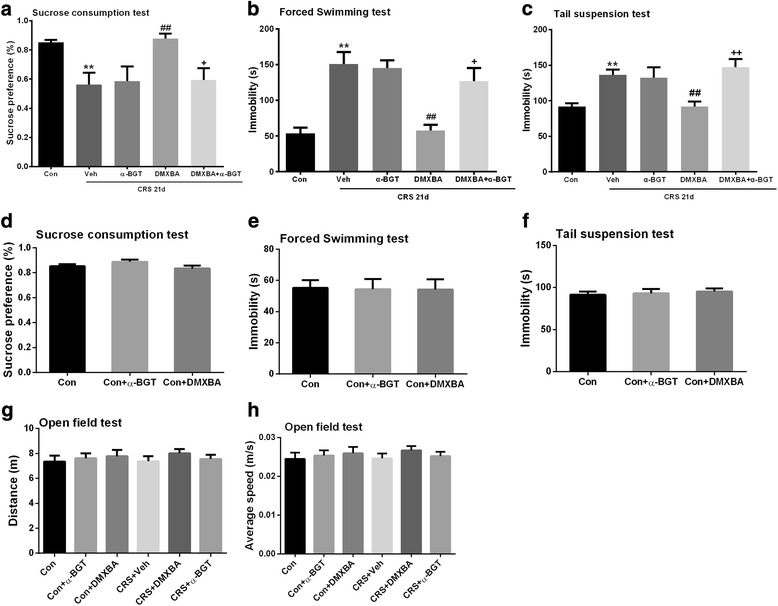
DMXBA treatment alleviates chronic restraint stress (CRS)-induced depressive-like behaviors. Mice were exposed to CRS for 21 days and treated with vehicle (Veh), DMXBA, or DMXBA plus α-bungarotoxin (α-BGT) beginning on day 10. **a**–**c** DMXBA treatment increased sucrose consumption in the sucrose preference test (**a**) and decreased immobility time in the forced swim test (**b**) and tail suspension test (**c**). Concurrent treatment with α-BGT reversed these effects. **d–h** Neither α-BGT nor DMXBA alone had an effect on depressive-like behaviors (**d**–**f**) or motor activity (**g**–**h**). Con, control group. Data are expressed as mean ± SEM. *n* = 27 per group; **P < 0.01 vs. control group; ^##^ *P* < 0.01 vs. CRS 21d + Veh group; ^+^ *P* < 0.05, ^++^ *P* < 0.01 vs. CRS 21d + DMXBA group

After 21 days of CRS, the mice showed significantly less sucrose consumption in the SPT, and spent significantly more time immobile in both the FST and TST (Fig. 4a–c). These results suggest that repeated restraint induces depression-like behaviors in mice. Notably, administration of the α7nAChR agonist DMXBA (4 mg/kg/d) beginning on day 10 of CRS restored the sucrose intake and reduced the duration of immobility in mice. However, concurrent administration of α7 nicotinic receptor antagonist α-BGT (1 μg/kg/d) with DMXBA eliminated the effects of DMXBA on depression-like behaviors, confirming the role of α7nAChR in mediating the effects of DMXBA. Neither DMXBA nor α-BGT had an effect on behavior of the mice that did not undergo CRS (Fig. 4d–f).

To rule out the possibility that the test drugs affected animal locomotor activity, we tested the spontaneous activity of one group of mice in the open field test. As shown in Fig. 4g–h, we found no significant difference in the total distance traveled or average speed between the treated and the untreated mice, indicating that CRS, DMXBA, and α-BGT had no effect on the locomotor activity of mice.

### DMXBA alleviates CRS-induced neuronal damage in hippocampus

**Fig. 5:**
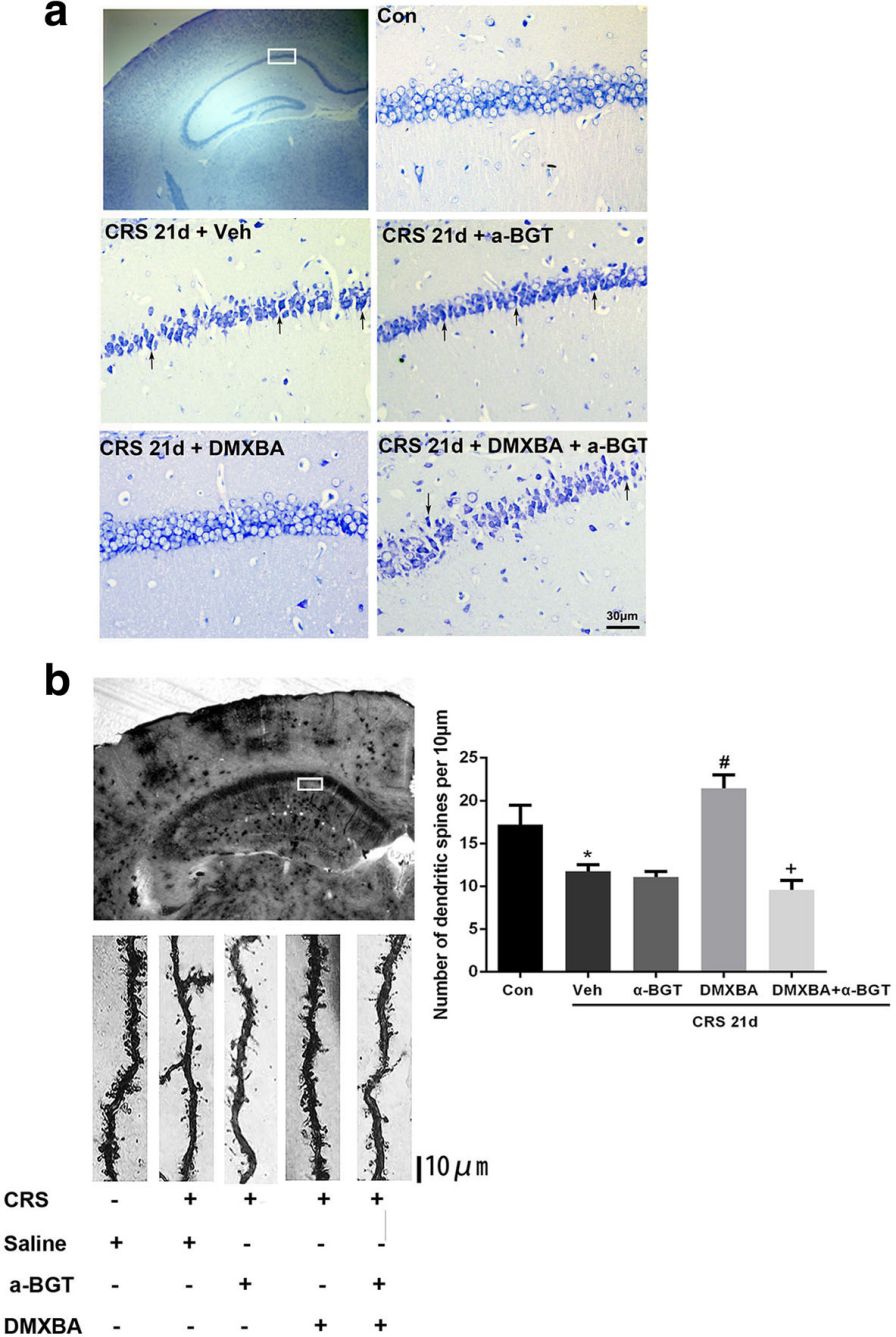
DMXBA treatment alleviates chronic restraint stress (CRS)-induced neuronal damage in the CA1 region of hippocampus. **a** Neuronal damage indicated by Nissl staining in the hippocampus of control (Con) and CRS mice treated with vehicle (Veh), DMXBA, or DMXBA plus α-bungarotoxin (α-BGT); scale bar, 30 μm. **b** Dendrite segments are revealed by Golgi staining in the CA1 region of hippocampus in the different groups; scale bar, 10 μm. Dendritic spines were counted on a 50-μm segment length in 10 neurons from each section. Con, control group. Data are presented as mean ± SEM. *n* = 6 per group; *P < 0.05 vs. control group; ^#^ *P* < 0.05 vs. CRS 21d + Veh group; ^+^ *P* < 0.05 vs. CRS 21d + DMXBA group

Neuronal damage in mouse hippocampal formations after CRS was estimated by Nissl and Golgi staining (Fig. 5). Nissl staining revealed hippocampal atrophy in the CRS mice. In the CA1 field of the hippocampus, neuronal damage in CRS mice was characterized by shrunken cell bodies and pyknotic nuclei (Fig. 5a, arrows). In contrast, mice treated with DMXBA beginning on day 10 of CRS had less neuronal damage and well preserved hippocampal morphology. Dendritic spine structure in the CA1 hippocampus was examined by Golgi staining (Fig. 5b). When we compared the hippocampal CA1 region of CRS and control mice, we observed significant dendritic spine loss in the CRS group. DMXBA treatment restored the dendritic spine density in stressed mice. Together, these results indicate that α7nAChR plays a critical role in maintaining neuron survival and dendritic spine integrity during chronic stress.

### DMXBA restores cholinergic signaling in hippocampus after CRS in mice

**Fig. 6:**
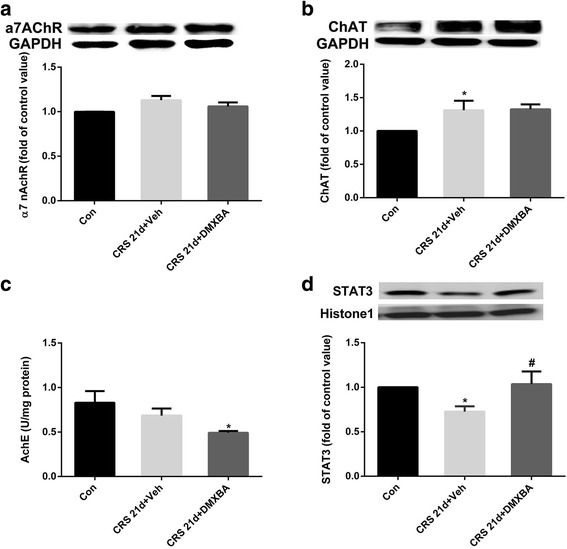
Chronic DMXBA treatment prevents chronic restraint stress (CRS)-induced cholinergic insufficiency. **a** Relative levels of α7nAChR protein in hippocampus. Results are shown as fold change relative to GAPDH protein level. **b** Representative immunoblots and relative levels of choline acetyltransferase (ChAT) protein in hippocampus. Results are shown as fold change relative to GAPDH protein level. **c** Acetylcholinesterase (AChE) activity in hippocampus of different groups. **d** Representative immunoblots and relative levels of STAT3 protein in hippocampus. Data are shown as fold change relative to histone protein level. Con, control group. Data are presented as mean ± SEM. *n* = 8 per group; *P < 0.05 vs. control group; ^#^ *P* < 0.05 vs. CRS 21d + Veh group

Because CRS altered central cholinergic signaling, we examined whether DMXBA regulated components of it. As shown in Fig. 6, DMXBA treatment did not alter the expression of α7nAChR or ChAT in the hippocampus of CRS mice. However, it did significantly reduce hippocampal AchE activity. In addition, DMXBA treatment significantly increased the expression of the nuclear transcription factor STAT3. These data indicate that DMXBA restores cholinergic signal transduction through α7nAChR activation, which leads to nuclear STAT3 activation.

### DMXBA alleviates CRS-induced neuroinflammation

**Fig. 7:**
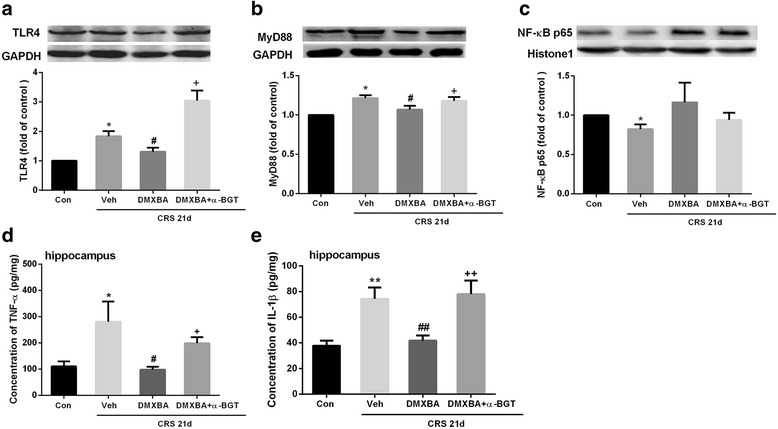
Chronic DMXBA treatment inhibits chronic restraint stress (CRS)-induced inflammatory signaling activation and proinflammatory cytokine over-expression. **a–c** Representative immunoblots and relative levels of TLR4 protein (**a**), MyD88 protein (**b**), and NF-κB protein (**c**) in hippocampus. Results are shown as fold change relative to GAPDH (**a**, **b**) or histone (**c**) protein level. **d** TNF-α levels in hippocampus by ELISA. **e** IL-1β levels in hippocampus by ELISA. Con, control group. Data are expressed as mean ± SEM. *n* = 8 per group; *P < 0.05, **P < 0.01 vs. control group; ^#^ *P* < 0.05, ^##^ *P* < 0.01 vs. CRS 21d + Veh group; ^+^ *P* < 0.05, ^++^ *P* < 0.01 vs. CRS 21d + DMXBA group

Considering that CRS elicited an inflammatory response characterized by increased TLR4 signal activation, we examined whether DMXBA was able to interact with elements of the TLR4 signaling pathway. As shown in Fig. 7, DMXBA treatment significantly decreased the expression of TLR4 and MyD88 in CRS mice, but did not alter the expression of NF-κB. DMXBA also inhibited CRS-induced activation of microglia and astrocytes. Consistently, TNF-α and IL-1β level in the hippocampus were significantly lower in DMXBA-treated mice than in vehicle-treated mice at 21 days after CRS. Moreover, concurrent administration of the α7 nicotinic receptor antagonist α-BGT (1 μg/kg/d) to CRS mice eliminated the effects of DMXBA on neuroinflammation, confirming the role of α7nAChR in mediating the effects of DMXBA. However, DMXBA treatment did not affect the serum levels of TNF-α and IL-1β (Additional file 1).

### DMXBA reverses CRS-induced changes to microglia-specific Iba-1 in mouse hippocampus

**Fig. 8:**
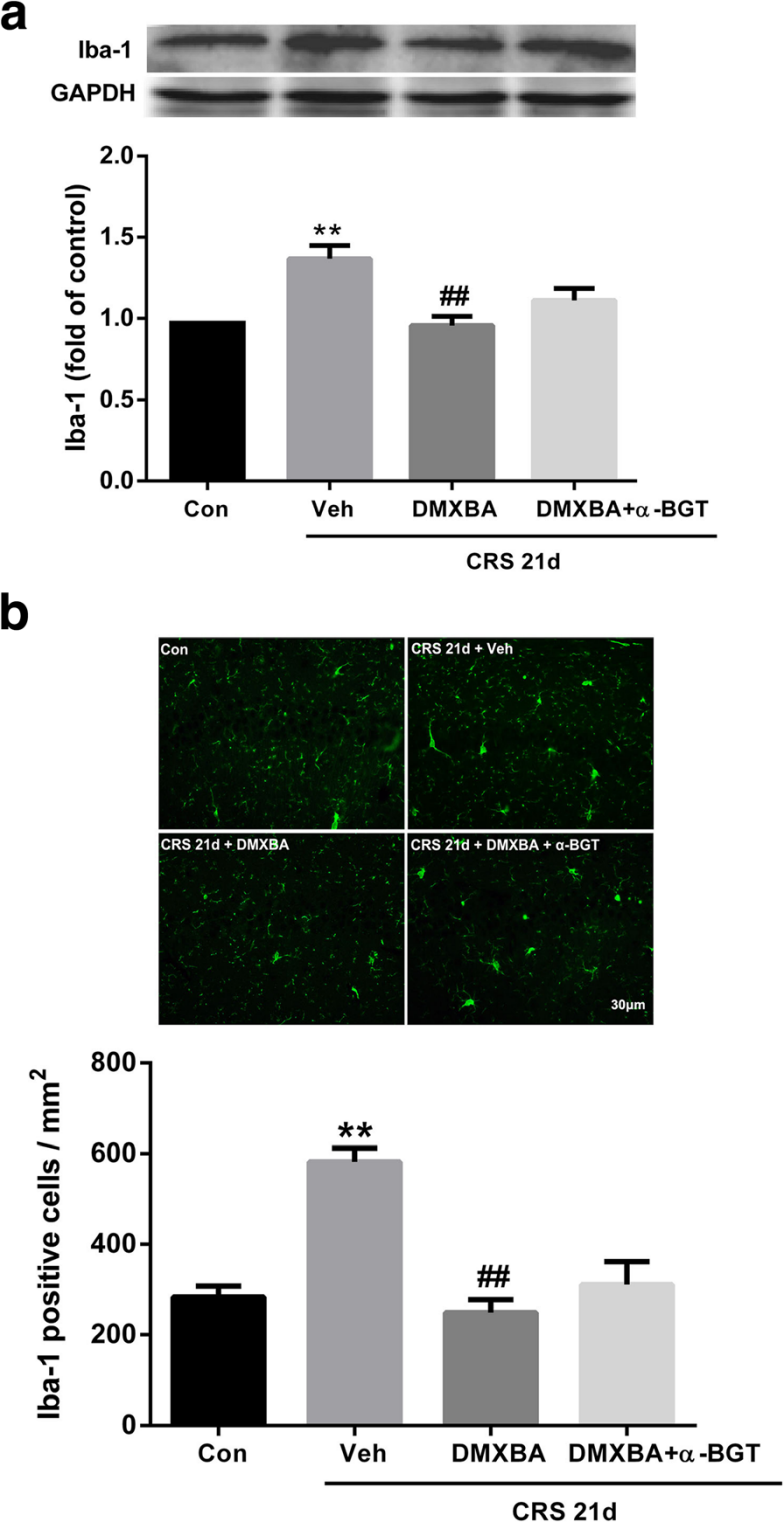
Chronic restraint stress (CRS)-induced microglial activation in mouse hippocampus is reversed by chronic DMXBA treatment. **a** Representative immunoblots and relative levels of Iba-1 protein in hippocampus. Results are shown as fold change relative to GAPDH protein level. **b** Iba-1 immunofluorescent staining and quantification analysis of positive cells in hippocampal sections. Scale bar, 30 μm. Con, control group. All data are expressed as mean ± SEM. *n* = 8 for protein expression; *n* = 4 for immunofluorescence assay. **P < 0.01 vs. control group; ^##^ *P* < 0.01 vs. CRS 21d + Veh group

Treatment with DMXBA significantly decreased the expression of Iba-1 in the mouse hippocampus (Fig. 8a). Similarly, immunofluorescence analysis indicated that DMXBA treatment reduced the number of Iba-1-positive microglia (Fig. 8b). Simultaneous administration of α-BGT (1 μg/kg/d) with DMXBA to CRS mice eliminated the effects of DMXBA on microglial inhibition, confirming the role of α7nAChR in mediating the effects of DMXBA.

### DMXBA relieves the decrease in Treg cell population in CRS mice

**Fig. 9:**
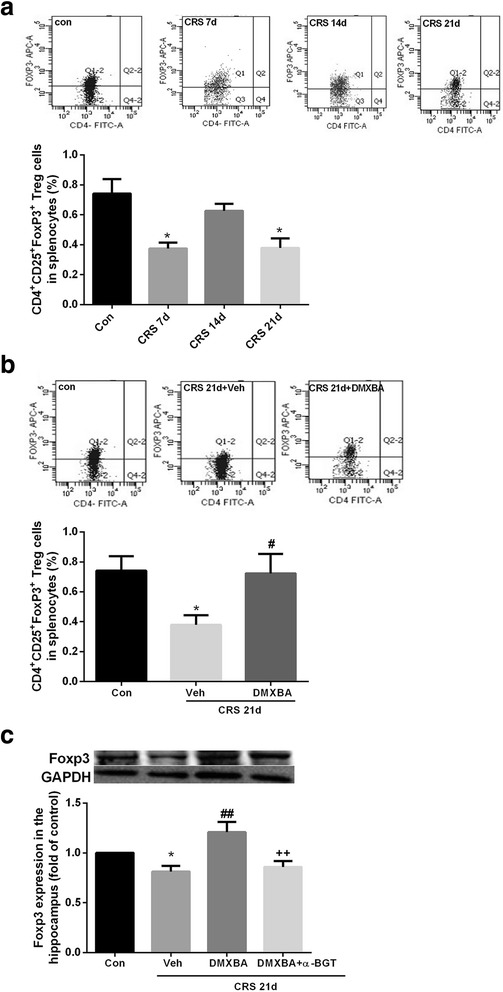
Chronic DMXBA treatment reverses chronic restraint stress (CRS)-induced reductions in T regulatory (Treg) cells in mouse periphery and hippocampus. **a** Representative flow cytometry profiles and statistics of Foxp3^+^-expressing cells among CD4^+^CD25^+^ splenocytes on days 7, 14, and 21 of CRS. **b** Representative flow cytometry profiles and statistics of Treg cells among splenocytes from mice treated with vehicle or DMXBA. **c** Representative immunoblots and relative levels of Foxp3 protein in hippocampus. Results are shown as fold change relative to GAPDH protein level. Con, control group. All data are expressed as mean ± SEM. *n* = 8; *P < 0.05 vs. control group; ^#^ *P* < 0.05, ^##^ *P* < 0.01 vs. CRS 21d + Veh group; ^++^ *P* < 0.01 vs. CRS 21d + DMXBA group

CD4^+^CD25^+^Foxp3^+^ Treg cells appear to modulate cerebral inflammation induced by pathogens and injuries and provide an endogenous protective function. We examined the proportions of Treg cells in the periphery by flow cytometry during the progress of depression. CRS for 21 consecutive days induced a significant decrease in Tregs after a transient rise, compared with numbers in the control group (Fig. 9a). Daily treatment with DMXBA beginning on day 10 of CRS significantly increased the Treg cell population (Fig. 9b). We then determined the expression of Treg marker protein FoxP3 in the hippocampus by western blotting. FoxP3 expression in the hippocampus was higher in DMXBA-treated mice than in vehicle-treated mice after 21 days of CRS (Fig. 9c). Simultaneous administration of α-BGT with DMXBA eliminated the effects of DMXBA on peripheral and central CD4^+^CD25^+^Foxp3^+^ Tregs, confirming the role of α7nAChR in mediating the effects of DMXBA.

## Discussion

The CRS model is known to induce psychological stress and is often used to study depression [37, 38]. Although diverse factors contribute to the development of depression, recent research has suggested that neuroinflammation has a significant role in the pathogenesis of depressive disorders [39, 40]. In this study, we found that the α7nAChR agonist DMXBA reduced inflammation and alleviated anhedonia and depression-like behavior in mice subjected to CRS.

Proinflammatory cytokines and glial activation are thought to contribute to neuronal damage after various cerebral injuries [41–44]. Some investigators have reported that the TLR4 signaling pathway plays an important role in neuroinflammation after chronic stress exposure [2, 3, 45], and others have shown that TLR4-deficient mice subjected to subacute stress exhibit only a minor inflammatory response in brain tissue [17]. Our study shows that repeated restraint induced-psychological stress leads to increased levels of TNF-α and IL-1β, as well as elevated activity of microglia in the hippocampus of mice. The increased inflammatory response in the hippocampus may be partly related to upregulation of the TLR4 pathway, although we observed no obvious stimulation of NF-κB p65 subunit in the brain hippocampus after CRS. Chronic inflammatory stimulation may restrict NF-κB action, as has been reported in a chronic mild stress model in rats [2]. Furthermore, we found that the selective α7nAChR partial agonist DMXBA mitigated chronic stress-induced activation of the TLR4 signaling pathway while reducing depression-like behaviors in mice. It has been reported that DMXBA antagonizes α4β2 nAChRs in addition to serving as an agonist for α7nAChRs [46]. Notably, we found that pretreatment with α-BGT, a selective α7nAChR antagonist, abolished the anti-depressive and anti-inflammatory effects of DMXBA, suggesting that the protective effects of DMXBA are α7nAChR-dependent. Although α-BGT at the dose we used did not reverse DMXBA’s inhibitory effect on microglial activation, some studies have reported that microglial α7nAChR does participate in the α7nAChR-mediated anti-inflammatory effect during ischemic events [15, 47, 48]. To our knowledge, these results provide the first evidence to support a regulatory role of the cholinergic system in chronic stress-induced behavioral deficits and the accompanying neuroinflammatory response in the hippocampus of mice.

The cholinergic system is important for maintaining proper central and peripheral immune and inflammatory responses and therefore is called the cholinergic anti-inflammatory pathway. This pathway modulates immune and inflammatory responses mainly through acetylcholine activation of α7nAChR [49]. The cholinergic anti-inflammatory pathway was shown to be effective in cerebral ischemia, intracerebral hemorrhage, and Alzheimer’s disease [14, 15]. In our study, we further demonstrated a role for the cholinergic system during chronic stress.

We found an upward trend of α7nAChR and a downward trend of AChE activity, as well as significant upregulation of ChAT expression and decreased nuclear STAT3 expression in the hippocampus after 21 days of CRS, indicating relative disturbance of the brain’s cholinergic function. Accompanied by significant neuronal loss/damage in the hippocampus after 21 days of CRS, increased expression of ChAT can be attributed to the smaller number of cells remaining. Hence, the trend toward an increase in α7nAChR may indicate a significant increase in the remaining neurons or activated glial cells. Recent human imaging studies have shown that acetylcholine levels are elevated throughout the brain in depressed patients [50, 51], indicating that hyperactivity of the brain’s cholinergic system might be a compensatory mechanism for controlling neuroinflammation in chronic stress. However, chronic stress-induced changes in the protein levels of ChAT and acetylcholine may cause maladaptive plasticity downstream of acetylcholine, such as dysregulation of JAK/STAT signaling, that can lead to symptoms related to depression. These observations in humans and animals have promoted interest in a proinflammatory/anti-inflammatory signaling balance hypothesis of depression.

It has been reported that α7nAChR activation interferes with other signaling pathways by activating JAK/STAT signaling and modulating gene transcription in immune cells [49, 52]. In peritoneal macrophages, the α7nAChR can recruit the tyrosine kinase Jak2, which activates STAT3 and subsequent signaling cascades. The Jak2-STAT3 pathway contributes to the anti-inflammatory potential of α7nAChR by inhibiting the release of anti-inflammatory cytokines. In our study, α7nAChR agonist DMXBA treatment significantly reversed CRS-induced downregulation of nuclear STAT3 in the hippocampus, indicating that pharmacologic activation of α7nAChR restores central cholinergic signaling function. In addition, DMXBA treatment inhibited AChE activity in the brain, an effect that might also promote the actions of central cholinergic signaling during CRS.

Tregs, a subpopulation of CD4^+^ T cells, play a crucial role in maintaining immune homoeostasis and preventing the development of many inflammatory diseases, such as rheumatoid arthritis, multiple sclerosis, and ischemic stroke. Although an increase in Treg cells in peripheral blood via signals from the sympathetic nervous system may contribute to stroke-induced immunosuppression [24], recent reports showed that systemic administration of purified Tregs suppressed inflammatory over-activation in the brain and attenuated neurovascular dysfunction after ischemic stroke [53, 54]. In contrast, Treg depletion exacerbated ischemic brain injury [55]. The neuroprotective effect of Tregs may relate in part to their ability to protect the blood–brain barrier and inhibit peripheral inflammatory cell infiltration into the brain [53, 54]. Recently, Kim et al. [56] reported that chronically stressed mice with Treg depletion exhibited reduced depression-like behaviors, indicating that Tregs might be associated with the pathophysiologic mechanism of chronic stress-induced depression. In our present study, we found that 21 days of CRS produced a decrease in the mouse splenocyte Treg population, consistent with previous reports of low Treg cell counts in the peripheral blood of patients with major depression [57, 58]. Interestingly, we observed that 7 days of CRS caused a decrease in Treg cells in mouse spleen, whereas 14 days of CRS restored the Treg population. Treg cells declined again after 21 days of CRS, possibly indicating a long-lasting and decompensated immunologic alteration after chronic stress. We found that treatment with α7nAChR agonist DMXBA completely reversed the chronic stress-induced decline in Treg cells and alleviated depression-like behaviors in CRS mice, indicating that α7nAChR activation may modulate depressive behavior by promoting Treg cell function, which in turn mitigates chronic stress-induced neuroinflammation.

Chronic stress has been shown to induce peripheral immunosuppressive effects and is thought to increase susceptibility to many diseases [59]. In our study, after 7 days of CRS, mice exhibited significantly higher serum IL-1β and TNF-α levels. However, after 14 and 21 days, these levels had declined and returned to the normal range. Interestingly, IL-1β and TNF-α levels were elevated in the hippocampus at 21 days of CRS, indicating unsynchronized changes of cytokines in the periphery and brain. Although the blood–brain barrier might restrict the passage of harmful factors into the brain in the early stage of injury, or the CNS might change from a steady state to an unstable state after long-term stress, the actual reason for temporal and spatial changes of IL-1β and TNF-α is not yet clear. Treatment with the α7nAChR agonist DMXBA significantly increased the Treg population in the circulation and reduced inflammatory over-activation in the brain but had no apparent effect on serum IL-1β or TNF-α level. Thus, it seems that α7nAChR activation restricts inflammatory response in the brain but does not exacerbate immunosuppression after chronic stress. These findings are in line with its regulation of immune homeostasis between the brain and periphery during chronic stress. A single acute stress and chronic repeated stresses may mobilize different pathophysiologic mechanisms, as a recent report showed that activation of the cholinergic system by acetylcholine or AChE inhibitor physostigmine increased anxiety- and mood-related behaviors in the TST and FST [60]; however, the detailed mechanism is not fully understood.

## Conclusion

In conclusion, our study showed that chronic repeated stress induced over-activated neuroinflammation in mice with depressive-like behavior. This response was accompanied by over-activation of TLR4 signaling and dysfunction of cholinergic anti-inflammatory signaling. Additional activation of the cholinergic system by α7nAChR agonist suppressed this stress-induced neuroinflammatory response and mitigated the stress-induced depression. Activation of α7nAChR signaling was associated with decreased TLR4 signaling, suggesting that a rebalance of α7nAChR and TLR4 signaling could be the underlying mechanism of action.

## Additional file

Additional file 1: Supplementary materials. **S1**. No significant alterations of TNF-α and IL-1β were found in the hippocampus at days 7 and 14 of CRS. **S2**. DMXBA treatment did not have any effect on the serum levels of TNF-α and IL-1β. (DOCX 195 kb)

## Abbreviations

AChE: Acetylcholinesterase
ChAT: Choline acetyltransferase
CNS: Central nervous system
CRS: Chronic restraint stress
FST: Forced swim test
IL-1β: Interleukin-1β
MyD88: Myeloid differentiation factor 88
NF-κB: Nuclear factor kappa B
SPT: Sucrose preference test
STAT3: Signal transducer and activator of transcription 3
TLR4: Toll-like receptor-4
TNF-α: Tumor necrosis factor-α
TST: Tail suspension test
α7nAChR: Alpha7 nicotinic acetylcholine receptor
α-BGT: α-bungarotoxin
