# Correction to: Pharmacologic activation of cholinergic alpha7 nicotinic receptors mitigates depressive-like behavior in a mouse model of chronic stress

**DOI:** 10.1186/s12974-022-02577-w

**Published:** 2022-09-15

**Authors:** Dan Zhao, Xulin Xu, Linna Pan, Wei Zhu, Xiaopei Fu, Lianjun Guo, Qing Lu, Jian Wang

**Affiliations:** 1 Department of Pharmacology, School of Basic Medicine, Tongji Medical College, Huazhong University of Science and Technology, Wuhan, 430030 China; 2 Department of Pharmacy, Jiangxi Provincial Cancer Hospital, Nanchang, 330029 China; 3 The Key Laboratory for Drug Target Research and Pharmacodynamic Evaluation of Hubei Province, Wuhan, 430030 China; 4 Department of Neurology, The Second Hospital of Nanchang, Nanchang, 330003 China; 5 Department of Emergency Medicine, Tongji Hospital, Tongji Medical College, Huazhong University of Science and Technology, Wuhan, 430030 People’s Republic of China; 6 Department of Anesthesiology and Critical Care Medicine, Johns Hopkins University, School of Medicine, Baltimore, MD 21205 USA; 7 Department of Human Anatomy, Basic Medical College of Zhengzhou University, Zhengzhou, Henan 450001 People’s Republic of China

## Correction to: Journal of Neuroinflammation (2017) 14:234 https://doi.org/10.1186/s12974-017-1007-2

Following the publication of the original article [1], it was noted that the authors identified an error in Figs. 2 and 9.

1. The representative western blot image for α7AchR protein expression in Fig. 2b was incorrect by mistake. The corrected version of Fig. 2 (JPG file) is given below.
2. CRS21d image in Fig. 9a, CRS 21d + DMXBA image in Fig. 9b are incorrect. During the preparation of figures, we inadvertently provided an incorrect image, and are updated with correct images, Con group image in Fig. 9a was also updated for more accuracy. The corrected version of Fig. 9 is given below.
3. We would like to add in the correction that "the authors clarify that this work was not financially supported by the NIH grants or by the Johns Hopkins University."

**Fig. 2:**
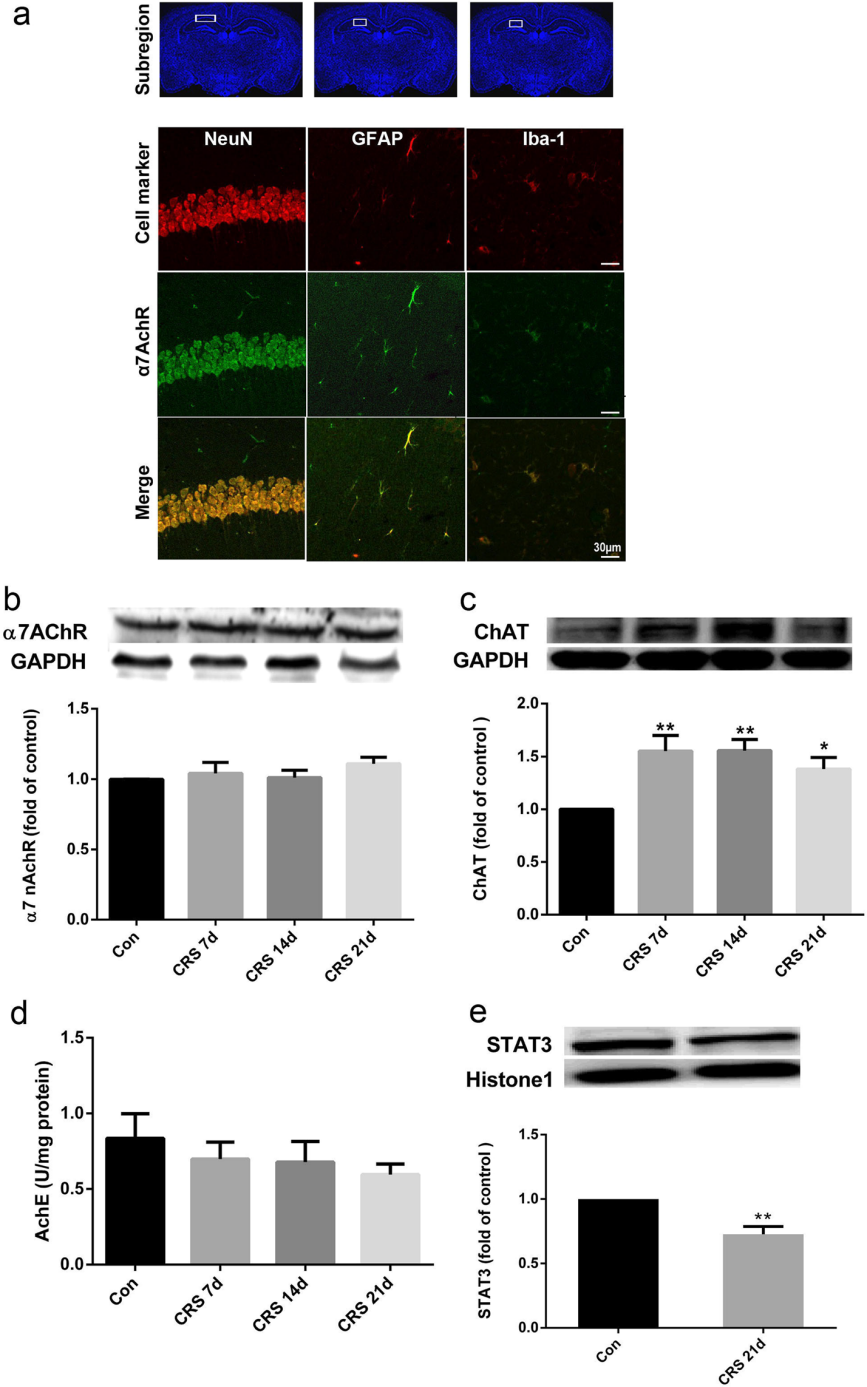
Chronic restraint stress (CRS)-induced alterations in components of central cholinergic signaling in hippocampus. **a** Doubleimmunolabeling showed that α7nAChR colocalized primarily with NeuN^+^ neurons and to a lesser extent with GFAP + astrocytes and Iba-1^+^ microglia in the hippocampus; scale bar, 30 μm. **b** Representative immunoblots and relative levels of α7nAChR protein in hippocampus. Results are shown as fold change relative to GAPDH protein level. **c** Representative immunoblots and relative levels of choline acetyltransferase (ChAT) protein in hippocampus. Results are shown as fold change relative to GAPDH protein level. **d** Acetylcholinesterase (AChE) activity in hippocampus at different time points of CRS. **e** Representative immunoblots and relative levels of STAT3 protein in hippocampus. Results are shown as fold change relative to histone1 protein level. Con, control group. Data are expressed as mean ± SEM. *n* = 8 per group; *P < 0.05, **P < 0.01 vs. control group

**Fig. 9:**
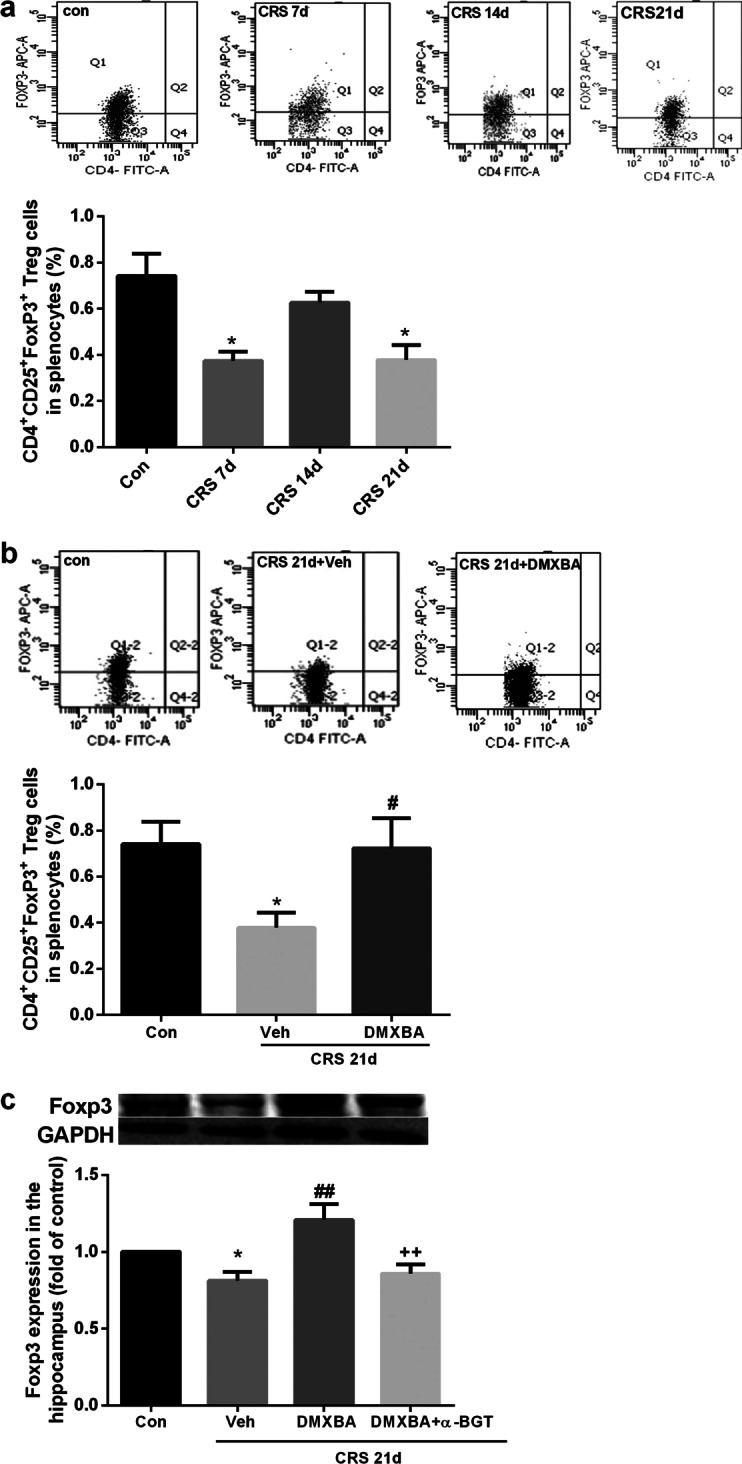
Chronic DMXBA treatment reverses chronic restraint stress (CRS)-induced reductions in T regulatory (Treg) cells in mouse periphery and hippocampus. **a** Representative flow cytometry profiles and statistics of Foxp3^+^-expressing cells among CD4^+^ CD25^+^ splenocytes on days 7, 14, and 21 of CRS. **b** Representative flow cytometry profiles and statistics of Treg cells among splenocytes from mice treated with vehicle or DMXBA. **c** Representative immunoblots and relative levels of Foxp3 protein in hippocampus. Results are shown as fold change relative to GAPDH protein level. Con, control group. All data are expressed as mean ± SEM. *n* = 8; *P < 0.05 vs. control group; ^#^*P* < 0.05, ^##^*P* < 0.01 vs. CRS 21d + Veh group; ^++^*P* < 0.01 vs. CRS 21d + DMXBA group

The corrections did not affect the overall conclusions and we hope to make the corrigendum as soon as possible. We apologize for this mistake due to our carelessness.
