# Supplementary material for: Pharmacologic activation of cholinergic alpha7 nicotinic receptors mitigates depressive-like behavior in a mouse model of chronic stress
Source: J Neuroinflammation. 2017 Dec 2;14:234. doi: 10.1186/s12974-017-1007-2 (PMC5712092; doi:10.1186/s12974-017-1007-2)
Supplement: Supplementary file 1 — Supplementary materials. S1. No significant alterations of TNF-α and IL-1β were found in the hippocampus at days 7 and 14 of CRS. S2. DMXBA treatment did not have any effect on the serum levels of TNF-α and IL-1β. (DOCX 195 kb) [file 12974_2017_1007_MOESM1_ESM.docx]

**Supplementary Materials**

**
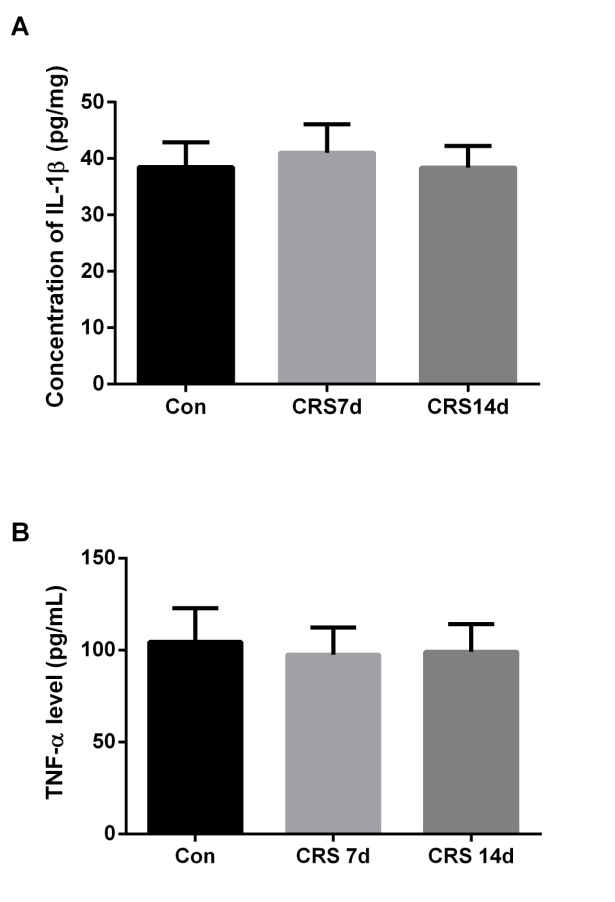

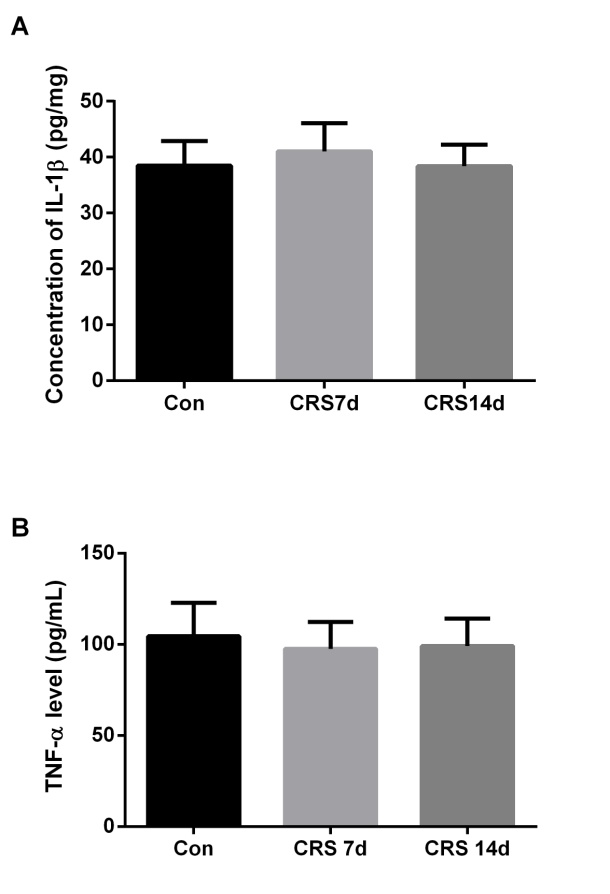
**

**S1. No significant alterations of TNF-α and IL-1β were found in the hippocampus at days 7 and 14 of CRS.** **All data are expressed as mean ± SEM. n = 8 per group.**


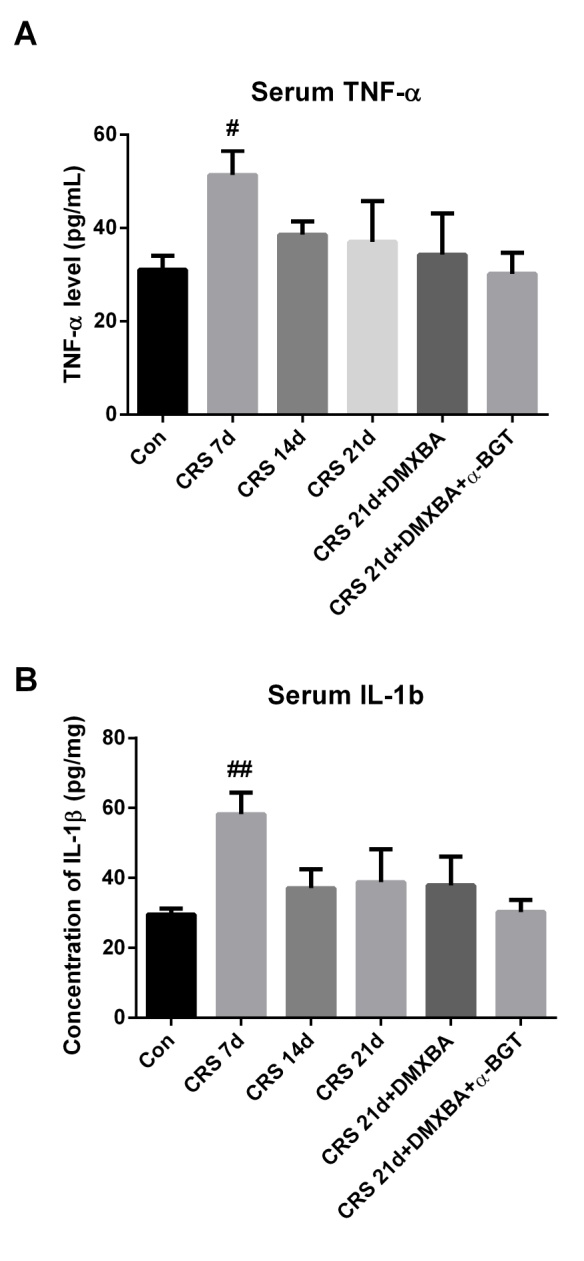

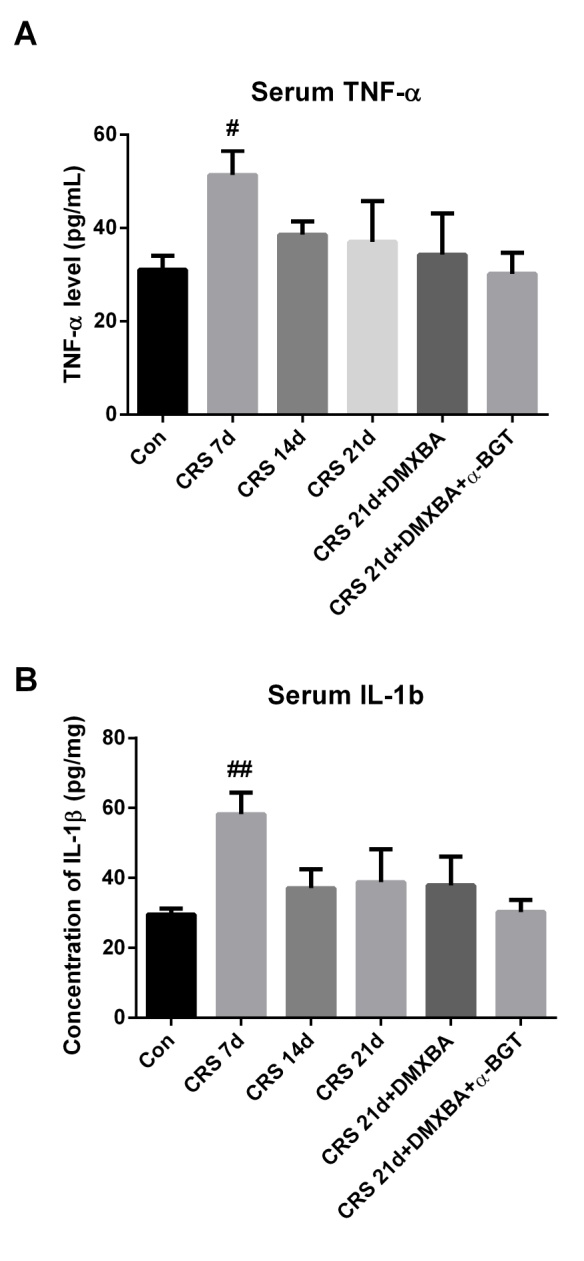


**S2. DMXBA treatment did not have any effect on the serum levels of TNF-α and IL-1β. CRS for 7d induced increased levels of TNF-α and IL-1β in the serum, however, CRS for 14d and 21d did not have any effect on the cytokines in the serum. DMXBA treatment did not alter the levels of TNF-α and IL-1β in the serum after CRS 21d. All data are expressed as mean ± SEM. n = 8 per group; ^#^P < 0.05, ^##^P < 0.01 vs. control group.**
